# Evaluation of Novel Co-Processed Excipients for Direct Compression Using the Sediment Delivery Model (SeDeM) Expert System

**DOI:** 10.3390/ph19091481

**Published:** 2026-09-17

**Authors:** Adriana Ciurba, Paula Antonoaea, Emőke Margit Rédai, Andrada Pintea, Cezara Pintea, Amalia-Adina Cojocariu, Magdalena Bîrsan, Mădălina-Florentina Mihalcea, Robert-Alexandru Vlad

**Affiliations:** 1 Pharmaceutical Technology and Cosmetology Department, Faculty of Pharmacy, George Emil Palade University of Medicine, Pharmacy, Science and Technology of Targu Mures, 38th Gheorghe Marinescu Street, 540142 Targu Mures, Romania; 2 Grigore T. Popa University of Medicine and Pharmacy from Iaşi, 16 Universităţii Street, 700115 Iaşi, Romania

**Keywords:** co-processed excipient, granules, binder, SeDeM expert system, powder layering onto starter cores

## Abstract

**Background/Objectives**: The development of multifaceted excipients is a requirement of the pharmaceutical industry. This study aimed to develop a granular co-processed excipient for tablets and to evaluate it using the SeDeM expert system. **Methods**: Four granule formulations were developed via the powder layering technique, varying binder concentrations (15% and 20%) and filler types (microcrystalline cellulose and lactose), using sugar as the core. The granules obtained were evaluated utilising the SeDeM expert system. The unloaded granules were compressed to yield uncoated compacted granules, which were verified for dimensional parameters, mechanical properties, and disintegration ability. **Results**: Varying binder concentrations (15% and 20%) together with differences in filler/disintegrant composition were associated with changes in particle-size distribution. During the SeDeM evaluation, E3 formulation exhibited good results in terms of parameter index (PI = 0.83), parameter profile index (PPI = 7.64), and Good Compressibility Index (GCI = 7.28). The recorded disintegration times were below 15 min for all compacted granules and complied with the Ph. Eur. 12 requirements for uncoated tablets. **Conclusions**: For granule development, variations in formulation composition, including binder concentration, were associated with differences in particle size and lubricity. The SeDeM expert system can be used in the preformulation studies to characterize powders or granule formulations from which uncoated tablets can be obtained, but there are several pharmacotechnical properties not included in the list of parameters that need to be analysed to comply with the industrial requirements.

## 1. Introduction

Single-component excipients do not always provide the necessary compressibility and tablettability to enable the formulation or manufacture of uncoated tablets [1]. Pharmaceutical excipients are pharmacologically inert substances used during manufacturing to protect, support, or enhance stability and bioavailability, and to aid in product identification [1].

As regulatory requirements for the purity, safety, and standardization of excipients have become stricter, the International Pharmaceutical Excipients Council (IPEC) has emerged, which defines a co-processed excipient as a combination of two or more excipients intended to physically modify their properties in a way that cannot be achieved by simple physical mixing and without significant chemical modification [1]. The growing need for efficient direct-compression formulations has increased interest in multifunctional co-processed excipients. By combining the advantages of different materials in a single system, these excipients can improve flowability, compressibility, and overall tablet-manufacturing performance [1,2].

In all cases, the resulting excipients are characterized in terms of quality. One of the tools that can be used in this case scenario to evaluate the granular excipients is the Sediment Delivery Model (SeDeM) expert system, a methodology applied in drug preformulation and formulation studies, especially in the case of solid dosage forms (uncoated tablets made through direct compression) [2,3]. Besides this particular use, this mathematical tool can be used to compare different excipients from the same category (different superdisintegrants, different functional excipients, different fillers) or with the same chemical formula (different types of lactose or microcrystalline cellulose) or to characterize the same excipient from different batches [4].

The main use of this expert system is to develop uncoated tablets by characterising active ingredients and excipients. This system provides information on the physical profile of the active pharmaceutical ingredient (API) and excipients. The SeDeM expert system highlights the advantages and disadvantages of APIs and excipients, indicating whether direct compression is appropriate. This system can be used as a screening tool in order to design the formulation in the final product [5,6,7].

Through the SeDeM system, 12 parameters are evaluated, each of which is included in five distinct incidence factors:

- Dimension (Bulk density (D_a_) and tapped density (D_t_),
- Compressibility (porosity—I_e_, Carr index—CI, Cohesion index—I_cd_),
- Flowability (Hausner ratio—HR, Angle of repose—α, Flowability—t″),
- Lubrication/stability (loss on drying—%HR, hygroscopicity—%H),
- Dosage/lubrication (particles < 160 µm—%Pf, Homogeneity index—Iθ) [2,4,6,8,9].

The main aim of this expert system, as stated above, is to improve time management and the properties of compacted granules (better mechanical properties). Firstly, the active ingredient is characterized using the SeDeM expert system, followed by screening the excipients (usually fillers or co-processed excipients), for which the most important factor of incidence is compressibility, as expected, since the compacted granules are developed through direct compression.

Khan has used this SeDeM expert system to evaluate the granules. As expected, differences occur for the following parameters (Pf and Iθ), for which sieves with a higher mesh size are required [9].

Until now, this expert system and other SeDeM-derived tools have been used primarily to characterise powders or powder mixtures of excipients already marketed by various pharmaceutical companies. Several active ingredients have shown compressibility issues, including ibuprofen, memantine, and cannabidiol [8,10,11].

As a result, this study focuses on developing a granular functional excipient.

Although SeDeM was initially conceived as a preformulation and material characterization instrument, numerous investigations have broadened its application towards the development and refinement of innovative co-processed excipients for direct compression. Illustrative examples include starch–microcrystalline cellulose composites (Salim et al., 2021) [2], rice starch-based multifunctional excipients (Trisopon et al., 2021) [12], and spray-dried mannitol–pregelatinized rice starch systems (Saokham et al., 2025) [13]. In all three cases, the developed excipients were powders; in the study conducted by Salim et al. [2], the co-processed excipient was developed via co-dispersion, whereas in the latter two, the excipients were prepared by spray-drying [12,13]. The co-processed excipients developed in this study were manufactured by powder layering onto starter cores, an approach that generates spherical granular excipients rather than powder-based systems.

The SeDeM Expert System constitutes a valuable preliminary formulation and screening instrument that can mitigate development duration and assist in predicting compatibility with direct compression [7,14]. Nonetheless, it should serve as a decision-support tool rather than an exclusive solution and should be complemented with experimental validation, stability assessments, and more comprehensive methodologies.

The SeDeM Expert System has notable limitations to consider when developing pharmaceutical formulations. While it effectively assesses powder properties and predicts suitability for direct compression, it primarily focuses on physical attributes such as flowability, compressibility, density, and moisture content [14,15]. It does not comprehensively evaluate critical factors such as active ingredient–excipient compatibility, chemical stability, dissolution behavior, or bioavailability. Additionally, its predictions are derived from mathematical indices, which may not fully capture the complexities of large-scale manufacturing [15]. Since the system depends heavily on the accuracy of experimental data, measurement errors can lead to inaccurate conclusions. Moreover, SeDeM was primarily designed for direct compression and may be less suitable for complex dosage forms or manufacturing processes. Therefore, it should serve as a preliminary screening and decision-support tool rather than a standalone method. Its results should be validated with compatibility studies, stability tests, dissolution testing, and pilot-scale production [2,7,15,16].

This study aims to develop a new co-processed excipient by preparing granules via the layering technique and to compare formulations developed with a modified SeDeM expert system to determine which granules are most suitable for uncoated tablet production. Until now, Khan (2019) has used this expert system to analyze granular formulations with ribavirin, modifying methods to meet formulation requirements for dosage and lubrication (homogeneity index and particles < 160 μm) [9]. In summary, while SeDeM has been widely used as a preformulation tool, its primary function has been the characterization and selection of excipients, rather than the systematic development of novel co-processed materials [3,10,17,18]. Previous investigations rarely concentrate on the optimization of excipient ratios or provide structured methodologies for property enhancement [2,12,13]. This study advances the field by applying SeDeM as a formulation-screening and decision-support tool.

Another aspect targeted in this study was to produce granules that meet flow, compressibility, particle-size, and stability requirements and function as a versatile excipient for standard-release tablets. If the granules are compacted, the resulting compacted granules must also meet criteria such as friability, mechanical strength, and disintegration. It is expected that differences in excipient type and concentration may influence granule-size distribution and consequently affect the physical attributes of the granular excipient and the resulting tablet quality.

During our research, the developed excipients were compared with other functional excipients, both granular and powder, to highlight the improvements achieved in this study. The proposed compositions exhibited improved compressibility and flow-related properties compared with several literature-reported co-processed excipients [2,12,13]. Limitations and future directions include the absence of a formal Design of Experiments (DoE) and potential multicollinearity among SeDeM indices, which future investigations should address.

## 2. Results and Discussion

Granulation yields were above 80% in all cases: 87.62% (E1), 88.95% (E2), 93.65% (E3), and 90.12% (E4).

The excipients, quantities, and concentrations used to prepare the granules were selected based on results from previous studies and our own tests to ensure compliance with the pharmacopoeial requirements in force.

Four granule formulations were developed in this study, and their evaluation will be further underscored. First, the granules were evaluated for quality using the SeDeM expert system, followed by evaluation of the compacted granules obtained through direct compression of the unloaded granules. This section explores how formulation variation was reflected in the SeDeM profile.

The binder was used in this way because, at concentrations over 20%, the granules became larger than anticipated. Consequently, binder concentrations of 15% or 20% were chosen. Although the amount of AquaPolish^®^STA added to the formulations was adjusted, the excipient’s composition remained constant. The variable studied was the amount of the functional excipient, not its composition.

### 2.1. Evaluation of the Granules by Means of the SeDeM-Specific Incidence Factors

This subchapter presents the results for the parameters evaluated by the SeDeM expert system, grouped into five distinct incidence factors (Table 1). To facilitate understanding, this subchapter discusses these results in five subchapters, each examining all formulations.

It is important to interpret the comparison between these results and SeDeM data for conventional powder excipients with caution. The materials developed here were produced by layering powders onto starter cores, yielding larger, spherical granules. These particle traits are known to enhance flowability, dosing performance, and compressibility indicators. As a result, the higher lubricity/dosage and GCI values may be due not only to co-processing effects but also to the inherent benefits of the granular structure. Therefore, these findings should be seen as indicative rather than directly equivalent to literature values for powder-based excipients.

The radius values reported in this study are software-derived morphological descriptors intended for comparative analysis and do not represent intrinsic physical material properties. Consequently, their interpretation in relation to compact material behavior should be considered qualitative rather than direct.

#### 2.1.1. Particle Size Incidence Factor

The radius values for D_a_ range from 5.2 (E3) to 6.5 (E4), while the D_t_ values range from 5.7 (E3) to 7.2 (E4). Table 1 presents the results for tapped and apparent density. As shown in Table 1, for all radii of D_a_ and D_t_, values higher than 5 were obtained, which is an advantage, since powders usually yield values lower than 5, especially for D_a_, as can be seen in the previously published studies [8,17].

Suñé-Negre et al. highlighted powder excipients with D_a_ radius values lower than 5: Pearlitol 200 SD, Tri-cafos, Prosolv HD90, Iso malt 721, Pharmaburst C1, Erysta [15]. All the excipients mentioned are powdered materials or co-processed powder blends suitable for direct compression. Khan et al. showed similar results for the radii of D_a_ and D_t_ of the developed granular excipients [9]. In conclusion, there are excipients on the pharmaceutical market that do not comply with the SeDeM expert system requirements for the particle-size incidence factor. Still, they are certainly exhibiting other parameters that fall under the other four incidence factors. Developing granular excipients tends to improve the particle-size incidence factor, as outlined in this study and in the study conducted by Khan et al., in which this incidence factor exceeded 5 in almost all trials [9].

#### 2.1.2. Compressibility Incidence Factor

Of the three parameters assessed, porosity and Carr’s Index were calculated from previously obtained apparent and tapped density results using the formulas provided in the materials and methods chapter. The cohesion index was determined experimentally using tablets produced by direct compression. As previously mentioned, if the resistance to crushing exceeded 200 N, a radius of 10 was assigned; this applied to E3, where the resistance to crushing was 209.8 N, resulting in a capped value of 10. Table 1 shows the results for these three parameters across the four formulations, along with the compressibility index. Although it was expected to achieve a compressibility incidence factor greater than 5, this was not observed due to low interparticle porosity and a low Carr Index.

Even if the porosity and the Carr index were low, the compressibility of the granules was not negatively affected. During the experiments, I_cd_ appeared to be one of the SeDeM parameters most closely associated with compressibility-related performance. One possible explanation for the higher I_cd_ values observed in formulations containing lactose and no sodium alginate is lactose’s brittle-fracture behaviour during compression, which can promote the formation of new bonding surfaces. However, because filler type and sodium alginate presence were not independently varied, this interpretation remains speculative, and their individual effects cannot be isolated. In addition, the increased value of I_cd_ may be attributed to the presence of the modified starch-based AquaPolish^®^ STA matrix, which is expected to exhibit deformation behaviour typical of polymeric binders and to promote interparticulate bonding during compaction [19,20].

Low interparticle porosity does not necessarily hinder compressibility because tablet formation depends on particle rearrangement, deformation, and bonding during compression. While high-porosity powders enable better rearrangement and densification, low-porosity materials can still show excellent compactibility if they have strong cohesion and plastic deformation.

Although low porosity and a low Carr index indicate limited particle rearrangement, compressibility was not adversely affected because plastic deformation and interparticulate bonding contributed. The binder may have facilitated deformation under compression, thereby increasing contact area and interparticulate bonding. However, this interpretation remains hypothetical and was not directly investigated in the present study. I_cd_ appears to be the most discriminatory compressibility-related parameter in the present study.

Similar results for this incidence factor were also observed in the article published by Khan et al., where compressibility ranged from 1.41 to 5.28, indicating low porosity, and the Carr Index led to these reduced values. This behaviour was observed in almost all of the outlined trials, with the exception of trial 10, where the I_cd_ was 0 (intermediate screw speed (25 rpm) and lowest roller speed (8 rpm) [9]. In conclusion, the low porosity and Carr Index values, along with the increased I_cd_, might be characteristic features of the granules.

#### 2.1.3. Flow Property Evaluation

As shown in Table 1, all the formulations exhibited high flowability incidence factor values, ranging from 8.25 (E2) to 9.54 (E4). For all three parameters used in calculating the flowability incidence factor, values > 5 were recorded, resulting in a high average. In addition, all four formulations specified RH = 10, while the latter three showed an extremely good flow factor and, as a result, were assigned a radius of 10. Even with very small α values, the radius values for this parameter were not at their maximum; nonetheless, the results supported the imposed average value, which is limited to at least 5.

One of the properties that influences the direct compression process is the flow capacity of the powder blend or mixture, as poor properties can lead to issues during the scale-up process or even in the preformulation or formulation stage when the eccentric press might experience hopper bridging, funnel clogging, and irregular powder descent, which can interrupt manufacturing. Other issues that might occur include inconsistent die filling (increased weight variation), capping, lamination, sticking, and picking [21].

#### 2.1.4. Evaluation of Lubricity/Dosage

To calculate the homogeneity index, particle distributions for the four formulations were analysed. The results for the two parameters characterising lubricity/dosage are presented in Table 1. The last two formulations achieved the highest values for both the homogeneity index and Pf; as a result, the lubricity/dosage incidence factor received the highest score of 10. For the first two granular formulations, the Iθ values were 10, while the P_f_ ranged from 7.66 (E1) to 9.17 (E2).

Khan et al., outlined similar results in terms of lubricity/dosage evaluation, with low values outlined for the active ingredient (<5), while for the granules loaded with ribavirin, the incidence factor showed the highest value comparable with the result obtained in this study (9.13—Trial 1—25 rpm—screw speed and 12 rpm—roller speed), which means that by increasing the particle size the lubricity/dosage incidence factor tends to show improved radius values compared to the lower dimensioned particles (powders) [9,17,22].

#### 2.1.5. Lubricity/Stability Evaluation

As shown in Table 1, all granular excipients had radius values greater than 5, ranging from 7.07 (E3) to 9.29 (E4) for HR. Conversely, E1 granular excipients did not exceed the proposed radius of 5, with a value of 4.62 for H. The E4 formulation showed the highest value for this parameter at 9.72. In summary, the mean incidence values ranged from 6.48 (E1) to 9.51 (E4).

These results are comparable to or superior to those mentioned in the literature for other granular mixtures or functional powders evaluated through SeDeM/SeDeM-derived expert systems, with Khan outlining a lubricity stability incidence factor between 6.46 (Trial 8, screw speed 15 rpm and roller speed 8 rpm) and 8.11 (Trial 2, screw speed 35 rpm, roller speed 8 rpm) [9,14]. Sune Negre and his collaborators reported values for this incidence factor ranging from 3.02 (Kollidon VA 64) to 6.95 (Isomalt 721) [15]. It can be concluded that increasing particle size improves the lubricity/stability incidence factor, a behaviour observed in both this study and Khan’s reported results [9].

### 2.2. Mathematical Evaluation of the Proposed Granular Excipients

**Figure 1:**
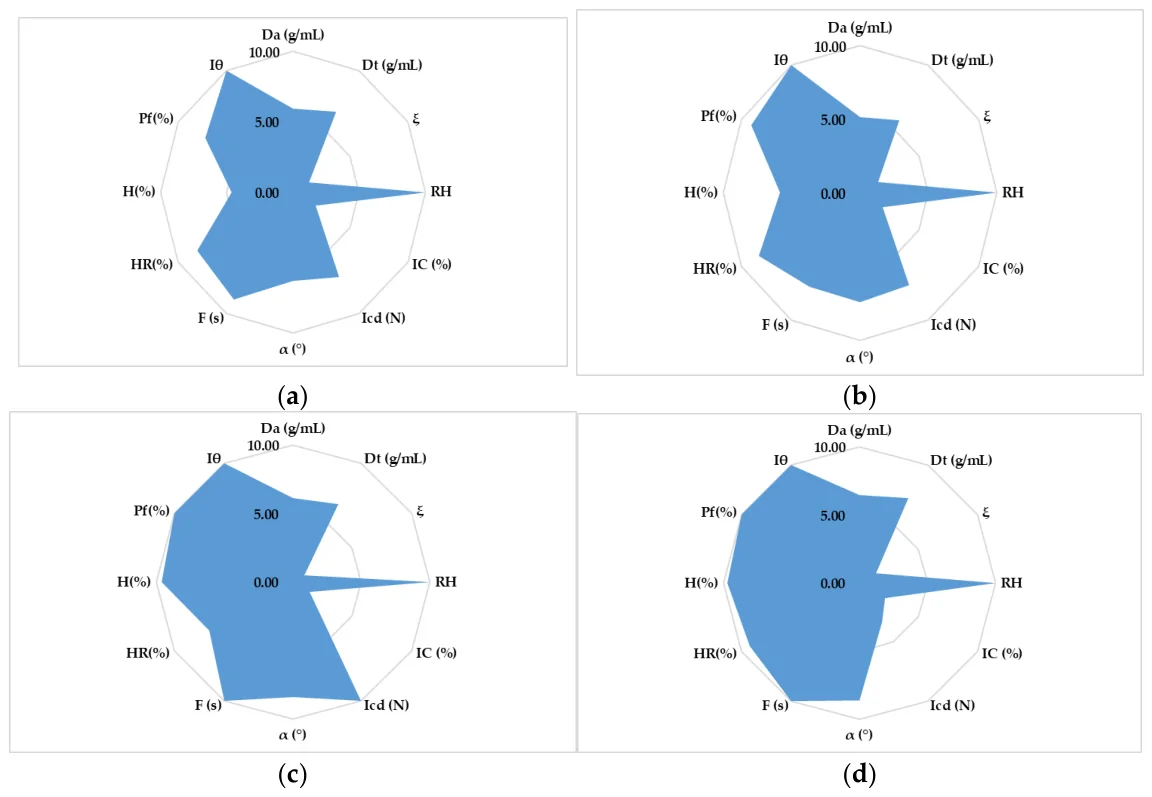
The SeDeM diagram for the developed granular excipients: E1 (**a**), E2 (**b**), E3 (**c**), E4 (**d**).

SeDeM diagrams were generated for the four granular excipients chosen for this study (Figure 1). Typically, a graphical evaluation can identify parameters that require adjustment, as well as those with radii close to 10. In practice, the greater the surface area encompassed by the combined radii of the 12 parameters obtained, the lower the likelihood of encountering problems during tablet development using the direct compression method (flowability, stability, compressibility, etc.). Out of the four granular functional excipients, E3 showed the lowest number of parameters that need improvement (two: porosity and Carr Index).

For example, if a star diagram is obtained, it indicates that several parameters tend to have high radii, while others tend to have extremely low values, which are not usually compatible with the selected compaction method discussed in this study. If the diagram shows all the parameters close to the maximum radius value (10), a dodecagon will be obtained, which is the hypothetical form of an excipient that can be achieved with the SeDeM expert system. This shape could be achieved by a perfect excipient that has been evaluated, for which all the radius values are 10. If all radii are 5, another dodecagon will be obtained, but its surface area will be lower; still, in this case, the minimum required radius will be obtained for all incidence factors. In practice, the surface obtained by tracing the SeDeM diagram represents the GCI.

These results are supported by the PI, PPI, and GCI indices, which will be discussed in the following subchapter.

**Figure 2:**
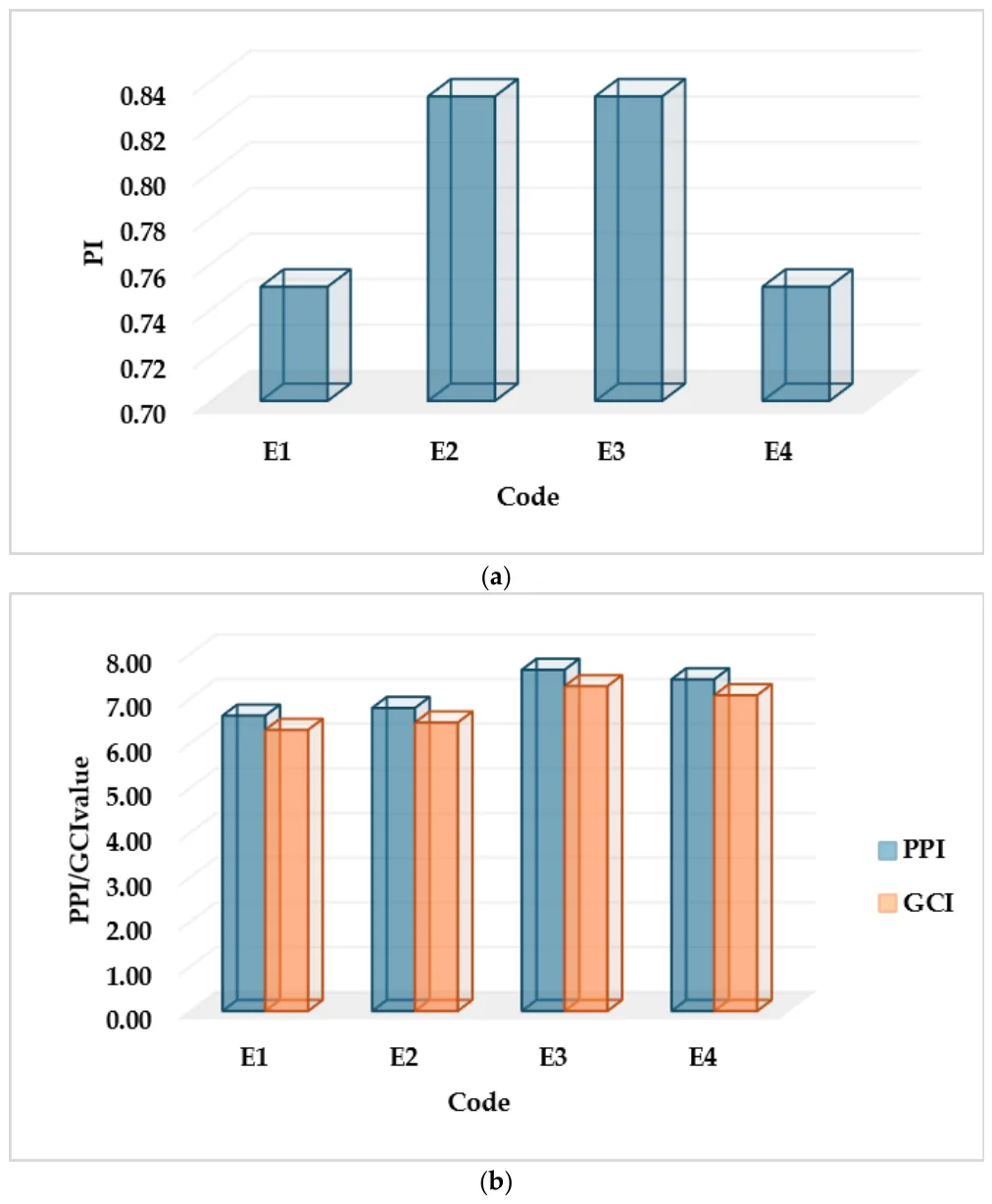
The PI (**a**), PPI, and GCI (**b**) indices obtained in the case of the developed granular excipients.

In the case of PI (Figure 2a, the granular excipients coded E2 and E3 exhibited the highest value of 0.83, while E1 and E4 showed a lower value of 0.75; even so, all the excipients developed are in the range recommended by the SeDeM expert system, 0.5–1 [14] (Table S1). In the study by Khan et al., PI values ranged from 0.4 to 0.67; in contrast, this research observed higher values for three formulations of granular excipients [9]. Sune Negre conducted research analysing several powders and functional excipients recommended for direct compression; the results show that none of the excipients had PI values higher than 0.5 [17].

PPI—a parameter that is calculated in the case of all SeDeM/SeDeM-derived expert systems—showed values that exceeded the limit value imposed by this mathematical tool (5) in the case of all four granular excipients proposed (Figure 2b). Because this parameter is used to calculate the GCI, the product of the PPI and a subunitary value, a higher PPI reduces the risk that the GCI will yield values that do not meet the SeDeM requirements. The PPI ranged between 6.62 (E1) and 7.64 (E3). Khan et al. reported lower values for this parameter (4.02 for the active ingredient, ribavirin) and 5.8 for loaded functional granules [9].

In the case of GCI, lower values were obtained than in the case of PPI, but even after comparing the results, it was observed that for all granular excipients (E1–E4), values higher than 5 were recorded (Figure 2b). Since the cohesion index (I_cd_) indicated good values for the E1–E4 granular excipients, it can be concluded that, for these excipients, a high GCI can be correlated with the compressibility incidence factor. Higher GCI values than those for powder excipients were obtained in this study [14,17,18].

The SeDeM expert system is commonly used during preformulation or formulation studies to find a suitable formulation or excipient that meets pharmacopoeial standards. In its application, granules are characterized based on various incident factors relevant to the scenario. GCI represents the normalised PPI based on the total number of factors evaluated (12), and it includes results for all parameters evaluated, not just those included in the compressibility incidence factor. As a result, in addition to evaluating the proposed indices, the values obtained for different incidence factors should be considered before starting the experiments.

According to the ICH Q8(R2) guideline (Pharmaceutical Development), material variability needs to be fully understood and controlled to ensure final product quality [23]. The SeDeM expert system used in this study serves as an upstream, proactive risk-mitigation tool within the QbD framework. By converting these 12 distinct parameters into radius values and then calculating several indices, the SeDeM diagram explicitly maps the Critical Material Attributes (CMAs) of the excipient matrix.

Integrating SeDeM data into an industrial Quality Risk Management framework (ICH Q9) enables manufacturers to predict tabletability and flow defects prior to compression, representing an important advantage, as predicting mixture behaviour requires knowledge of how the initial excipients behave [24].

### 2.3. Compacted Granules’ Quality Assessment

During tablet development, several parameters can pose challenges. The most important consideration is the compressibility of the active ingredient. In many cases, the active ingredient is not compressible; therefore, an excipient with good compressibility should be considered. Usually, when developing tablets, several excipients in varying amounts must be added to the formulation to comply with pharmacopoeial requirements for mechanical properties (resistance to crushing, crushing strength, friability), disintegration, and, finally, dissolution. By developing a ready-to-use excipient, the risk of obtaining tablets with low mechanical properties that require special storage due to the primary packaging is reduced. Using a functional excipient reduces the risk of low compressibility compared with non-co-processed excipients. If the disintegration time is short, the amount of disintegrant can be considered when comparing with other formulations.

As mentioned in the materials and methods section, this subchapter will be divided into several chapters to better outline the results. Firstly, the dimensional parameters (diameter and thickness) will be included, followed by the mechanical properties proposed in this study (friability, resistance to crushing, tensile strength, crushing strength/friability ratio), and, finally, the disintegration ability will be discussed.

#### 2.3.1. Dimensional Parameters (Thickness and Diameter)

**Figure 3:**
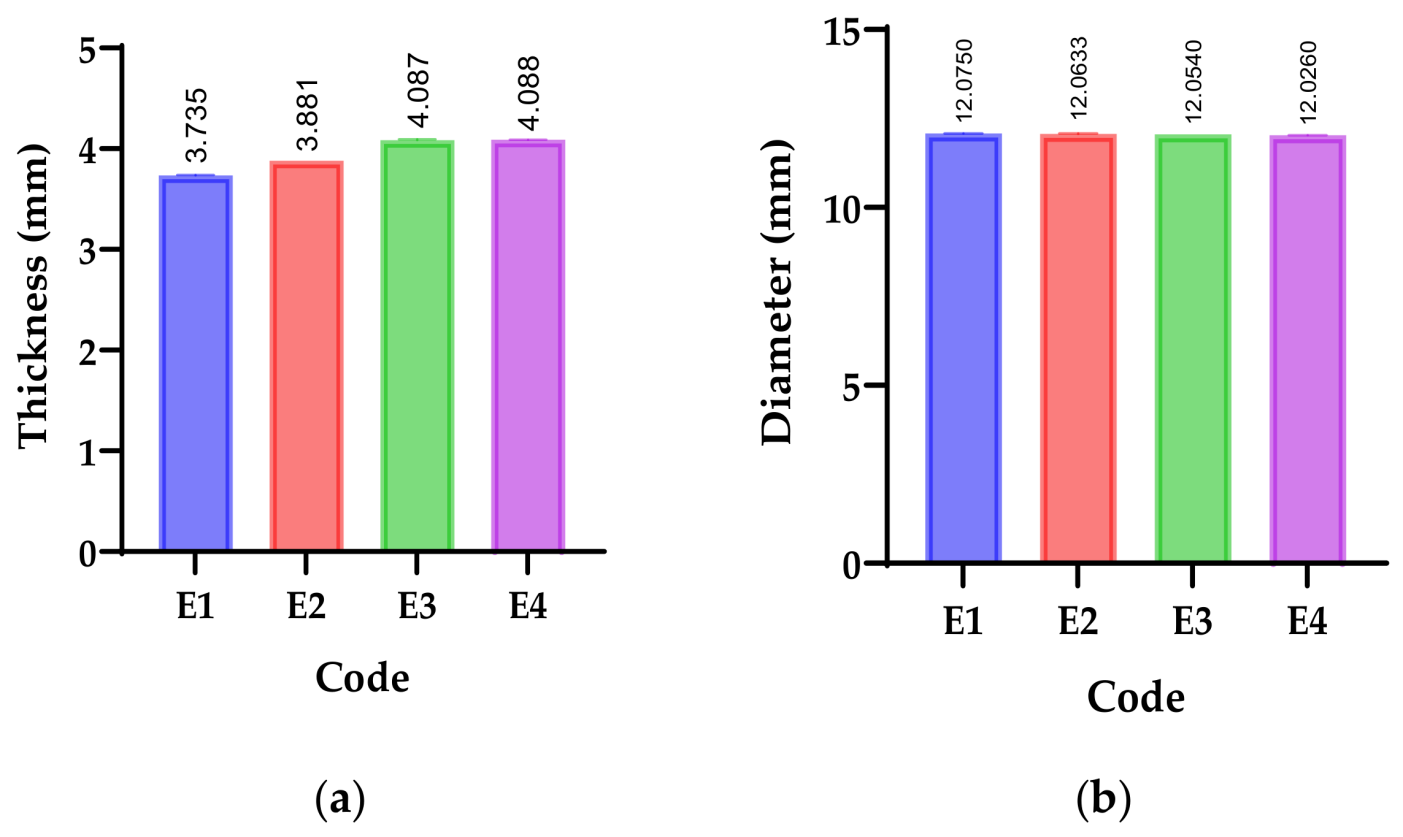
The dimensional parameters ± SD for the developed compacted granules’ thickness (**a**), diameter (**b**).

The results for the thickness and diameter are shown in Figure 3a and Figure 3b, respectively. The variation in thickness can be attributed to differences in interparticle porosity among the granules and the excipients used; for example, in E1 and E2, increasing the binder concentration increased thickness. Comparing formulations E1–E2 with E3–E4, the latter generally exhibited higher thickness values. However, because the comparison also involves the simultaneous absence of sodium alginate in E3–E4, the observed difference cannot be attributed solely to filler type [25].

The measured tablet thickness slightly exceeded the punch penetration depth, attributable to elastic recovery of the compact following decompression and die ejection—an occurrence frequently observed in pharmaceutical compaction procedures [25].

The observed thickness differences arise from formulation and compression variations, whereas tablet geometry remains consistent within each batch. The very low standard deviations and coefficients of variation (0.024–0.17%) indicate good process control and reproducibility during tablet compression (Table S2). Additionally, the narrow measurement ranges and stable mean values confirm uniform tablet dimensions across replicate tests. E3 and E4 had the greatest thickness, while E1 produced the thinnest tablets. Overall, the thickness data show that all formulations meet the criteria for dimensional consistency, which is crucial for uniform tablet weight, mechanical strength, packaging compatibility, and patient acceptance.

Minor differences were observed among the formulations in tablet diameter (Figure 3b), with all measured values remaining close to the target size (12 mm) (Table S3). The low standard deviations and very low coefficients of variation (ranging from 0.033% to 0.127%) reflect a high level of dimensional consistency and excellent control over the compression process. Additionally, the narrow ranges (0.008–0.030 mm) and tight confidence intervals confirm the reproducibility of tablet dimensions within each batch. Skewness values for E1, E3, and E4 were near zero, indicating symmetrical data distribution; E2 showed only slight positive skewness. Overall, the findings confirm that all formulations produced tablets with highly consistent diameters, meeting pharmaceutical quality standards for dimensional uniformity and ensuring reliable downstream processing, packaging, and patient acceptance.

#### 2.3.2. Mechanical Parameters

Since mechanical strength is a key requirement for tablet development, several parameters in this category were assessed: friability, resistance to crushing, tensile strength, and the crushing strength/friability ratio.

During compression, densification shifts from particle rearrangement to deformation-driven consolidation. While initial porosity is low, plastic deformation collapses voids and increases particle contact. This improves bonding and compensates for poor reorganization.

**Friability**

**Figure 4:**
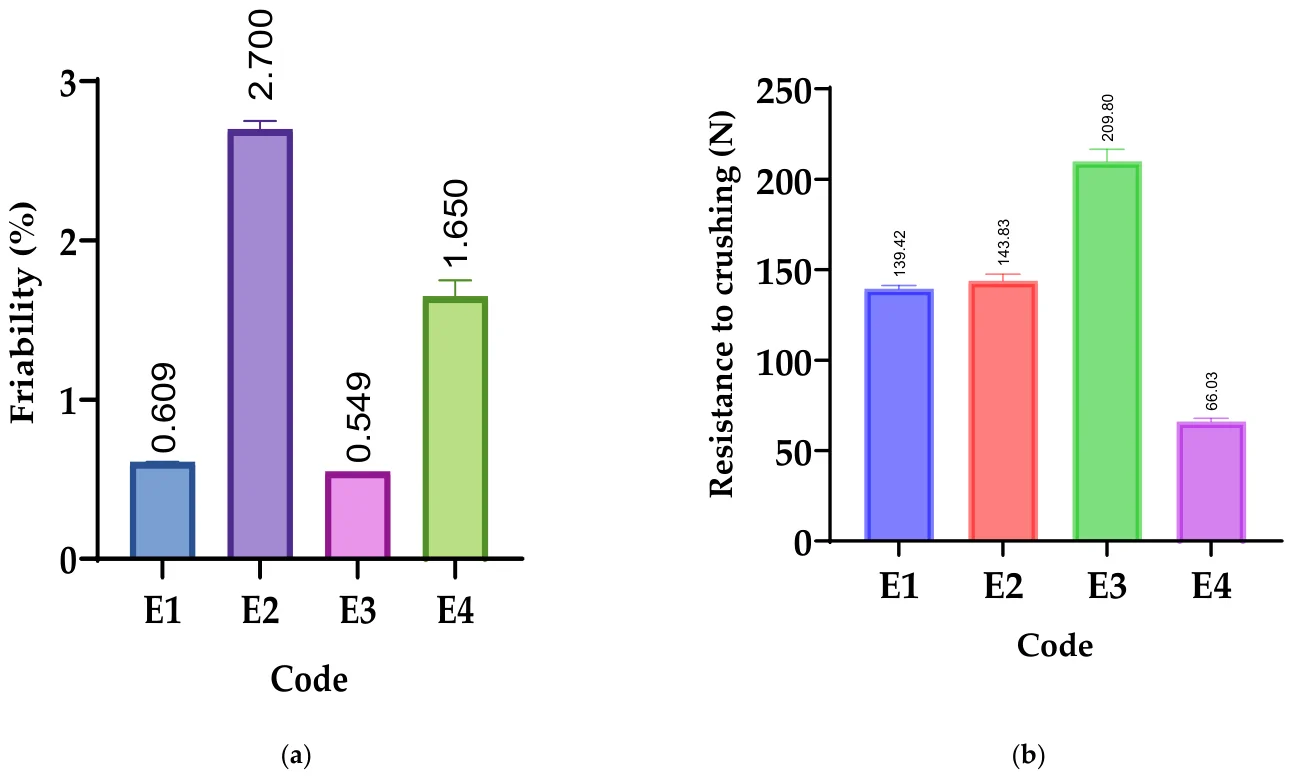

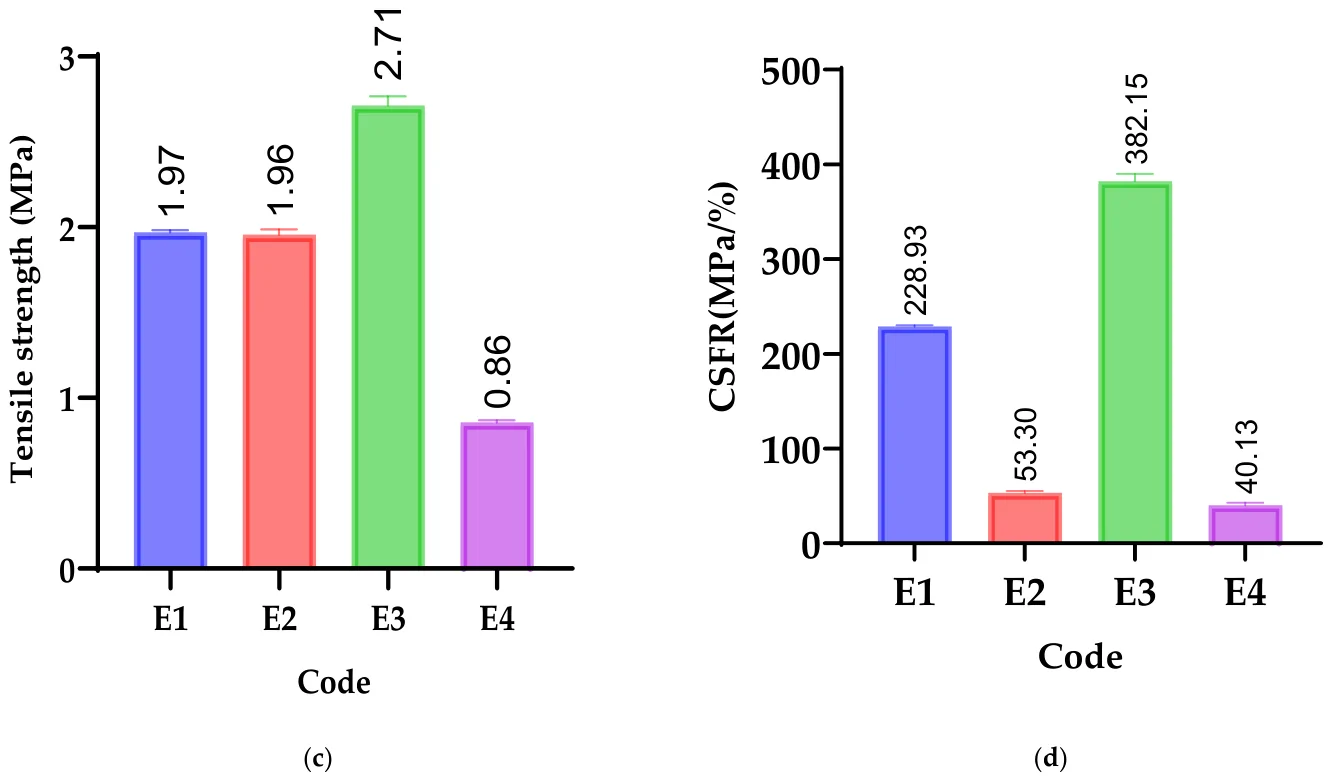
The results for: friability (n = 3) (**a**), resistance to crushing (n = 6) (**b**), tensile strength (**c**), and CSFR (**d**); Results are expressed as the mean ± SD.

The initial mass of 10 tablets and their final mass after processing were measured, and their friability was calculated. Of the four formulations developed, the following correspond to the requirements of Ph. Eur. 12 (E1 and E3), with values lower than 1%; two formulations (E2 and E4) stand out for exceeding the limit (>1%) (Figure 4a) [26]. The lower friability observed for E1 and E3, despite their lower binder content, suggests that friability was not governed solely by binder concentration. The results suggest that formulation composition, including filler type and sodium alginate presence, may have contributed to differences in friability. However, because these factors were varied simultaneously, the present experimental design cannot determine their individual contributions. These formulation factors likely affected coating structure and particle cohesion, resulting in lower friability for E1 and E3. The friability assessment showed excellent mechanical resistance, with mean friability values ranging from 0.549 ± 0.001% to 0.609 ± 0.001% for the first and third formulations, respectively. The low standard deviations and coefficients of variation (0.164–0.182%) observed for these formulations further confirm the high reproducibility of the manufacturing process (Table S4). The second and fourth formulations showed mean values of 2.70 ± 0.05 and 1.65 ± 0.10, respectively, exceeding the pharmacopoeial limit.

The higher performance observed in formulations containing 15% binder may be associated with a more favourable balance between particle cohesion and granule porosity; however, because binder concentration, filler type, and sodium alginate content varied simultaneously, the specific contribution of each factor cannot be determined from the present experimental design. At elevated concentrations, binders can enhance granule cohesion to the point that particle breakage is reduced, and the formation of new bonding surfaces during compaction is constrained [27,28]. Consequently, formulations containing 15% binder were associated with greater mechanical strength and improved disintegration properties. However, the specific contribution of binder concentration cannot be isolated from the effects of filler type and sodium alginate content within the current experimental design.

**Resistance to crushing**

In the case of resistance to crushing, the results increased in the following order: E4 < E1 < E2 < E3. Among the tested formulations, the lactose-containing systems were associated with higher crushing strength values than the microcrystalline-cellulose-containing systems. However, because filler type and sodium alginate presence were varied simultaneously, the individual contribution of either factor cannot be determined from the present experimental design. The compacted granules from E3 exhibited the highest crushing resistance, with values exceeding 200 N. Even so, at a binder concentration of 20%, the results for this mechanical parameter decreased dramatically, with the lowest value across all formulations recorded at 66.03 N (E4). The difference between E3 and E4 is most likely due to the increased concentration of AquaPolish^®^ STA and the slightly higher binder dispersion used in E4. Because the remaining formulation variables were unchanged, the behaviour in Figure 4b suggests that binder content played an important role in determining this parameter. However, further investigation is needed to confirm the exact mechanism underlying the observed difference.

All formulations showed low coefficients of variation (1.33–3.24%), indicating consistent intra-batch quality and reliable reproducibility in compression (Table S5). The narrow 95% confidence intervals and limited measurement ranges further validate the data’s dependability. Overall, the hardness results confirm that these formulations have sufficient mechanical strength to endure handling, packaging, and transportation stresses, with E3 yielding the best results among the tested batches.

By comparing the results with those in the literature, focusing on directly compressible compacted granules, the crushing strengths recorded for E3 fall within the range typically associated with good mechanical properties, whereas the compacted granules made with the granules coded E2 and E4 align more closely with intermediate-strength tablets.

**Tensile strength**

For tensile strength, compacted granules from granules coded E1 and E2 showed results close to 2 MPa, whereas for E4, the value was below 1 MPa. The highest value for this parameter was observed for E3, which correlates with its resistance to crushing, as the same formulation also exhibited the highest values.

Measurement variability was minimal, with coefficients of variation ranging from 0.74% to 2.03%, highlighting the consistency of the manufacturing process (Table S6). Additionally, the reliability of these results is supported by the narrow confidence intervals and small standard deviations. Overall, all formulations produced mechanically stable tablets, with E3 exhibiting the greatest resistance to fracture and the most favourable mechanical properties among the tested batches.

In a previous study in which orodispersible tablets containing drotaverine hydrochloride were developed, and the concentration and disintegrant type were varied, the highest value recorded for this parameter was 1.94 MPa [29]. Brniak et al. compared two formulations used to develop orodispersible tablets by varying the compression force and observed values close to 2 MPa at the intermediate compression level in both cases. In the formulation where Avicel PH 101 was used at the highest compression level, the highest tensile strength was observed (>8 MPa) [30].

Formulations E1-E3 demonstrate tensile strength values consistent with or above accepted literature thresholds (≈1–2 MPa), indicating adequate mechanical robustness.

**CSFR**

The CSFR is a parameter not included in pharmacopoeias. It has been discussed in several articles; however, because the way crushing strength is expressed varies across studies, comparisons are difficult. To address this, the tablet’s CSFR results are presented across different ranges, with the lower range corresponding to lower mechanical properties and the upper range to higher mechanical properties.

Formulation E3 underscored the highest CSFR, reflecting its optimal combination of high crushing strength and low friability. Conversely, E4 had the lowest ratio, indicating weaker mechanical performance (correlated with the high friability and lower resistance to crushing compared to E1–E3). The low coefficients of variation for E1 (0.55%), E2 (3.60%), and E3 (2.05%) highlight the good measurement reproducibility, whereas E4 shows slightly higher variability at 7.13%, which is still acceptable (Table S7). Additionally, narrow confidence intervals and limited data spread validate the reliability of these results. Overall, the CSFR ranking (E3 > E1 > E2 > E4) indicates that E3 has the strongest structural properties and the greatest resistance to mechanical stress.

The values can be grouped into three categories: <100 MPa/% for compacted granules E2 and E4; 200–300 MPa/% for compacted granules E1; and 300–400 MPa/% for compacted granules E3.

Considering the results obtained during the mechanical properties evaluation, it can be stated that the granules coded E1 and E3 would be better candidates for the development of compacted granules with different quantities of drug loads, while the other two formulations need some adjustments in order to comply with the Ph. Eur. 12 requirements regarding friability and automatically CSFR [26].

The high crushing strength observed for E3 also correlates with its high tensile strength and CSFR value, suggesting that this formulation provided a favourable balance between granule cohesiveness and interparticle bonding during tabletting.

Performance differences among E1–E4 stem from compaction mechanisms that affect interparticulate bonding. The tablet’s strength depends on solid bridges, mechanical interlocking, and intermolecular forces such as van der Waals forces and hydrogen bonds. Formulation E3 exhibited higher PI, PPI, and GCI, indicating greater true contact areas, stronger bonds, and improved compact integrity.

### 2.4. Tablet Disintegration Properties

**Figure 5:**
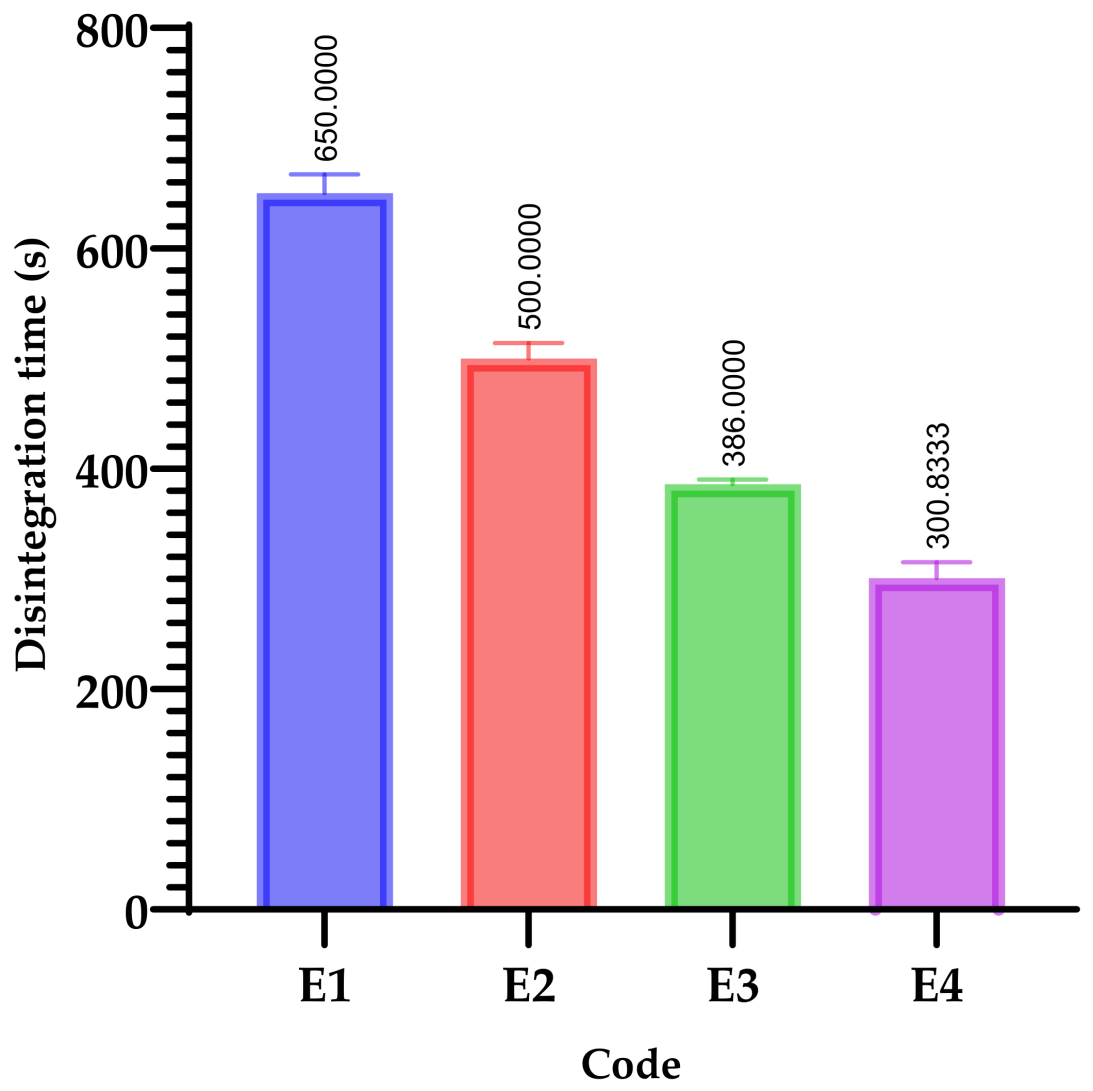
The evaluation of the disintegration ability ± SD (n = 6).

After the compacted granules were manufactured, the final test for quality evaluation was disintegration testing, with results reported in seconds. For compacted granules obtained by compressing the granules, a disintegration time of less than 11 min (660 s) was observed (Figure 5); as a result, the excipients might be used to develop uncoated tablets with varying drug loads. The disintegration time increased in the following order: E4 < E3 < E2 < E1, ranging between 300.833 (E4) and 650 s (E1). The results obtained are in accordance with the Ph. Eur. 12 (<15 min) stipulations, considering uncoated tablets [26]. All formulations showed low variability (CV < 5%), with E3 showing the highest precision (CV = 1.10%) (Table S8). The close agreement between mean and median values suggests the absence of substantial skewness, supporting the reliability and consistency of the experimental measurements.

Another comparison can be made between formulations with the same binder concentration to highlight the importance of sodium alginate. This comparison is somewhat difficult because the first two formulations used microcrystalline cellulose as the filler, whereas the latter two used lactose; as a result, two factors can be considered when discussing this aspect. In cases (E1–E3; E2–E4), disintegration time decreased, which may be attributed to a combination of factors (changing the filler and removing the disintegrant), as a disintegrant is usually associated with reduced disintegration time.

### 2.5. Regulatory and Industrial Relevance

Although the technical performance of a co-processed excipient (CoPE) is crucial, its commercial success and industry acceptance largely depend on a clear regulatory approval pathway. The CoPE developed in this research offers specific functional benefits—such as improved flowability and compactibility—that cannot be achieved through simple physical powder blending. To translate these multiple material benefits into a regulated industrial process, a defined strategy is necessary, including establishing its classification in pharmacopoeias and safety qualification.

According to IPEC and USP guidance, co-processed excipients are generally considered physical combinations of existing excipients, provided that the manufacturing process is not expected to induce new covalent bonds, generate novel impurities, or alter the fundamental chemical structures of the starting materials [1]. Accordingly, safety qualifications are exclusively centred on establishing batch-to-batch chemical equivalence and ensuring long-term physical stability [31,32].

For widespread industrial adoption, the long-term pathway involves securing a standalone compendial monograph within the USP-NF or Ph. Eur., mirroring existing monographs for established co-processed materials (e.g., Silicified Microcrystalline Cellulose) [26,31,32]. Until a dedicated monograph is published, the material can be effectively registered within a drug product dossier via a Type IV Drug Master File (DMF) in the United States or fully detailed within the marketing application dossier in the European Union, provided the manufacturing process is proven to be robust and reproducible [32,33].

## 3. Materials and Methods

Granules were prepared by powder layering onto starter cores using AquaPolish^®^ STA (a binder composed of hydroxypropyl methyl cellulose, hydroxypropyl cellulose, and other cellulose ethers, supplied by Biogrund GmbH, Hünstetten, Germany) at concentrations of 15% and 20%. Sugar (AGRANA Beteiligungs-AG, Vienna, Austria) was used as the core, yielding four formulations (E1–E4). The remaining excipients were sorbitol (Merck KGaA, Hesse, Germany) for its sweetening properties; sodium alginate (gifted by JRS Pharma GmbH & Co. KG, Rosenberg, Germany), which can serve as a disintegrant; sodium stearyl fumarate (Pruv^®^ sodium stearyl fumarate—JRS Pharma GmbH & Co. KG, Rosenberg, Germany), a lubricant; microcrystalline cellulose (Alfa Aesar, Ward Hill, MA, USA); and lactose (DFE Pharma, Goch, Germany), the latter two serving as fillers. Table 2 shows the quantities and roles of the components.

As illustrated in Table 2, two fillers were used: microcrystalline cellulose for formulations E1–E2 and lactose for E3–E4. The presence or absence of the disintegrant was evaluated, with sodium alginate used in the first two formulations and omitted from the latter two. Furthermore, the binder concentration was set to the following levels: 15% (E1, E3) and 20% (E2, E4).

### 3.1. Granules’ Manufacturing Steps

The powders were gradually added to a mortar in increasing amounts and ground until a fine, uniform powder was obtained (the powder-mixing process), resulting in 100 g of mixed powders (n = 1). The targeted yield was >80%. No wet massing occurred before the pan-layering process. The mixture was then divided into 10 equal portions. The blending was performed at laboratory scale. The procedure was applied consistently across all four formulations, though it was intended for preliminary screening.

The starter cores (⌀ = 0.5 mm) served only as substrates for particle build-up. Specifically, 20 g of sugar cores were layered with 100 g of excipient solids, yielding a core-to-layer ratio of 1:5 (*w*/*w*) and a batch size of 120 g. Because most of the particle mass and functionality originates from the integrated excipient layer rather than the inert starter core, the resulting material is considered consistent with the IPEC concept of a co-processed excipient.

After setting the pan at a 30° angle and 35 rpm, cores were added and humidified with binder. Every two minutes, powder was added in portions, with binder spray applied after each portion up to the tenth. Drying was then carried out at 40 °C for 24 h. Other important process parameters were binder solid content: 15–20%, and pan load used = 0.12 kg. Before evaluating the formed granules, sieving was performed using 2 mm and 800 µm mesh sizes. Granules retained on the 2 mm sieve were discarded, while those on the 800 µm sieve were further evaluated. Residual moisture content of the granules after drying was not measured, although all formulations were dried under identical conditions.

### 3.2. Evaluation of the Granules Obtained by Means of the SeDeM Expert System

The parameters outlined for the prepared granules define the SeDeM expert system. These twelve parameters are organised into five incidence factors and will be elaborated on in the following sections. The results are the average of three measurements for each formulation.

The equations are presented in the context of the SeDeM expert system. In this manuscript, all parameters used by the SeDeM expert system are characterised and described to ensure compliance with all steps required for using this mathematical tool. After the parameters are calculated, the equation is applied, the values are converted to radii, and the SeDeM diagram is traced.

#### 3.2.1. Particle Size

This incidence factor includes two parameters: D_a_ and Dt [34].

**Bulk density (D_a_) and Tapped density (D_t_)**

The European Pharmacopoeia 12th edition describes three methods for determining bulk volume and density. For practical use, the first method employed a 50 mL graduated cylinder containing granules with a pre-measured mass that occupied 60–70% of the volume. Each sample’s results are averages of three determinations [26]. Tapped density is measured by repeatedly tapping the powder in a graduated cylinder. After the initial volume or mass measurement, the cylinder is tapped mechanically, and volume readings are taken after 10, 50, 500, or 1250 tappings [26,34].

The bulk density was calculated using the following formula:

D_a_ = m/V_a_ (1)

where

m—weighed mass (g);

V_a_—bulk volume (mL).

The tapped density was calculated using the equation:

D_t_ = m/V_t_ (2)

where

m—weighed mass (g);

V_t_—tapped volume (mL).

The results were expressed as average and transformed into radius values by applying the SeDeM expert system stipulations (n = 3) [34].

#### 3.2.2. Compressibility

The three parameters considered to determine the compressibility parameter are: Porosity, Carr’s Index, and Cohesion Index.

**Porosity (ξ)**

Porosity (n = 3) is the first parameter included in the compressibility incidence factor, and it is calculated using the formula [10]:

(3) $$\xi \;=\frac{Dt-Da}{Da*Dt}$$

**Carr’s Index (CI)**

Carr’s index (n = 3), also called the compressibility factor [35], quantifies the compaction ability [36]. It is calculated using the bulk density and the tapped density using the equation:

(4) $$\mathrm{CI}=(1-\frac{Da}{Dt})\times 100$$

**Cohesion index (I_cd_)**

The cohesion index (n = 6) is calculated by compressing granules into 12-mm tablets with an eccentric press—TDP-6 (Shanghai Tianfeng Pharmaceutical Machinery Co., Ltd., Shanghai, China) and measuring crushing resistance (N) using a Tablet Four-Usage Tester—TFUT3 Biobase Biolin Co., Ltd., Shandong, China) [26].

#### 3.2.3. Flow Property Assessment

Parameters included in the flow incidence factor include the Hausner ratio (HR), angle of repose (α), and flow factor (t″) [35].

**Hausner’s Ratio (HR)**

The Hausner ratio (n = 3) is a measure of a powder’s ability to settle and assesses the importance of particle interactions [26,35]. It is calculated using the tapped density and the bulk density according to the equation below:

HR = D_t_/D_a_ (5)

**Angle of repose (α)**

α (n = 3) is the cone angle formed when granules pass through a funnel with set dimensions (top inner diameter: 140 mm, discharge orifice (outlet): 10 mm, funnel cone angle: 60°).

The funnel is positioned on a support 20 cm above the table, centred on a millimetre sheet. Its closed end contains the evaluated granules. The funnel connects to a vibrator (speed level = 10) starting at t = 0. The experiment ends when all granules are on the sheet, forming a heap or cone. The cone’s height (h), base radius (d/2), and flow time are measured [26,35].

This is calculated according to the formula:

tan α = h/r(6)

h—cone height (mm);

r—cone radius (mm).

Flow factor (t″)

The flow factor (n = 3) is the time, measured in seconds and tenths of a second (t), required for a preestablished amount of sample (m) to flow from the funnel onto a flat surface [14,22,25]. The formula used is as follows:

t″ = m/t (g/s)(7)

#### 3.2.4. Lubricity/Dosage Evaluation

To evaluate lubricating capacity and dosage, the following two factors are considered: the homogeneity index and the percentage of particles smaller than 160 µm.

**Evaluation of the Homogeneity Index (Iθ)**

This parameter follows Ph. Eur. 12 provisions. To measure particle size by the sieve test, 100 g of sample is vibrated for 10 min at a speed of 10 through sieves of 2500 µm, 800 µm, 315 µm, 200 µm, and 160 µm. The retained percentages on each sieve are calculated, along with the amount passing through the 160 µm sieve. The homogeneity index (n = 3) is then calculated using the specified equation. [9,34]:

(8) $$I\theta \;=\;\frac{Fm}{100+(\sum \left(di\times Fi\right)}$$

where

Iθ—Homogeneity index;

Fm = percentage of powder in the predominant (majority) size fraction

Fi = percentage retained in each of the other sieve fractions

di = distance (number of sieve intervals) between that fraction and the predominant fraction

**% Particles < 160 µm**

The determination of particles smaller than 160 µm (n = 3) is performed following the procedure described for establishing the homogeneity index. The quantity of granules passing through the 160 µm sieve is weighed [9].

#### 3.2.5. Lubricity/Stability Assessment

Loss on drying and hygroscopicity are parameters used to evaluate the lubricity and stability of the studied powders.

**Hygroscopicity (%H)**

%H (n = 3) was determined by calculating the weight gain after 1 g of granules was kept in a desiccator at an ambient humidity of 75% ± 2% and a temperature of 22 °C ± 2 °C for 24 and 72 h, respectively [8,36,37].

**Loss on drying (%RH)**

Loss on drying (n = 3) follows Ph. Eur. 12: drying samples at 105 °C ± 2 °C until constant mass in an oven. The temperature was chosen based on the melting point. 1 g of each formulation was spread onto Petri dishes, left for 30 min, and then weighed until the mass stabilised [8].

#### 3.2.6. Parameter Conversion into Radius Values

The conversion of the parameters included by the SeDeM expert system is underscored in Table 3. For all selected parameters, some factors are applied to obtain radius values in the range 0–10. For I_cd_, if the crushing resistance exceeds 200 N, a radius of 10 will be assigned.

#### 3.2.7. Acquiring the SeDeM Diagram

After obtaining the radius values for all 12 parameters, the SeDeM diagram is plotted as a radar chart in Microsoft Excel and visually evaluated to identify necessary alterations to address active-ingredient or excipient limitations. Next, the assessment of the Parameter Index (PI), Parameter Profile Index (PPI), and Good Compressibility Index (GCI) is performed, as detailed in the next subchapter.

#### 3.2.8. Assessment of the Parameter Index (PI), Parameter Profile Index (PPI) and Good Compressibility Index (GCI)

**Parameter Index (PI)**

This index measures the ratio of parameters with values exceeding 5 to the total number of parameters assessed by this expert system (12). Experts on this mathematical tool suggest that this value should be greater than 0.5.

**Parameter Profile Index (PPI)**

This parameter is the average value of all twelve radius values obtained. For this index, a value greater than 5 is recommended to comply with the SeDeM expert system’s stipulations.

**Good Compressibility Index (GCI)**

This index is specific to SeDeM and is calculated as follows (Equation (9)):

GCI = PPIxf,(9)

where

f—reliability factor and represents the surface occupied by the polygon inscribed in the circle, f = 0.952. For this specific index, a value > 5 is recommended.

### 3.3. Uncoated Compacted Granules Manufacturing Process

After characterizing the granules with the SeDeM expert system, they were compacted into uncoated tablets by direct compression with 12 mm punches at a compression pressure of 15 kN and a speed of 20 rpm on a TDP-6 tabletting machine. The tablets were then measured for diameter and thickness, and their mechanical properties—such as friability, crushing resistance, and disintegration—were evaluated.

### 3.4. Uncoated Tablet Quality Assessment

Quality is a fundamental requirement across all sectors and is also a primary objective within the pharmaceutical industry [38,39]. Given that uncoated compacted granules without active ingredients were developed (n = 50), certain traditional tablet assessments were considered [40].

#### 3.4.1. Determination of Tablet Diameter and Thickness

Dimensional parameters (thickness and diameter) were determined for 10 compacted granules from each sample using a digital micrometer (Yuzuki, India) [41] (accuracy 0.01 mm).

#### 3.4.2. Mechanical Property—Tablet Friability

To assess tablet friability, a TFUT3 apparatus (Biobase Biolin Co., Ltd., Shandong, China) was used, operated at 25 rpm for 4 min. For tablets with a unit weight of 650 mg or less, a sample comprising 10 entire tablets was utilised; for tablets weighing more than 650 mg, a sample of ten tablets was selected [26]. Before testing, the tablets should be carefully dusted. The 10 tablets were weighed accurately, with the initial mass (mi) recorded. The tablets were further rotated for 4 min at 25 ± 1 revolutions per minute, after which any dust was removed, and they were weighed again to determine the final mass (mf) [26].

The following formula was used to calculate friability:

F = (m_i_ − m_f_)/m_f_ × 100 (10)

where

F—friability (%);

m_i_—initial mass of the compacted granules;

m_f_—final mass of the compacted granules after dedusting.

#### 3.4.3. Mechanical Property—Resistance to Crushing

This test aims to determine, under defined conditions, the crushing strength of compacted granules, measured as the force required to break or fracture them.

To measure mechanical strength, the tablets were placed between two plates, and for the measurements, 10 compacted granules were used, ensuring that all fragments were removed before each test. The average of the 10 measurements was calculated and expressed in Newtons.

#### 3.4.4. Tensile Strength

Another physical parameter evaluated in the case of compacted granules is the tensile strength [42], which can be obtained by applying the equation:

Ts = (2 × F)/(π × d × h)(11)

where

Ts = tensile strength (MPa)

F = resistance to crushing (N)

d = compacted granules’ diameter (mm)

h = compacted granules’ thickness (mm) [43,44].

The results obtained are the average of six evaluations.

#### 3.4.5. Crushing Strength—Friability Ratio (CSFR)

CSFR is a metric used to evaluate tablet quality by integrating two previously determined parameters: tablet crushing strength and friability. This measure of physical strength is obtained by dividing crushing strength by friability (see Equation (12)). The influence of various types and quantities of disintegrants on the mechanical robustness of the tablets can be assessed by examining the CSFR value [30,44].

CSFR = Resistance to crushing/friability(12)

#### 3.4.6. Tablet Disintegration

This test determines whether compacted granules disintegrate within the specified time when placed in a liquid medium. Disintegration does not mean the complete dissolution of the unit or its active ingredient. Complete disintegration is defined as the state where any residue of the unit is a soft mass and has no palpable firm core [29].

The apparatus Biobase TFUT-3 tester (Tablet Four-Usage Tester, Biobase, Jinan, China) includes a basket-rack assembly, a 1 L beaker, a thermostatic device to heat the disintegration media to 37 ± 2 °C, and a component that moves the basket into the immersion fluid at a steady frequency of 29 to 32 cycles per minute [26].

The procedure involves inserting one tablet into each of the six basket tubes. The specified medium (600 mL selected because it ensured compliance with the required immersion depth and proper operation of the apparatus throughout the test) was maintained at 37 ± 2 °C and used as the immersion fluid. Upon completion of the designated time, the basket was withdrawn from the liquid, and the compacted granules were analyzed: all dosage units had fully disintegrated. If one or two dosage units did not disintegrate, the test was repeated with an additional twelve dosage units. The test requirements are met if at least 16 of the 18 dosage units disintegrate within 15 min [27,30].

### 3.5. Statistical Evaluation

Descriptive statistics were generated with GraphPad Prism 11.0.0 (Dotmatics, Boston, MA, USA). The data were summarized using the mean and standard deviation (Mean ± SD), median, minimum, maximum, and range. The coefficient of variation (CV%) was calculated to show the dataset’s relative variability. Additionally, the 95% confidence interval (95% CI) for the mean was calculated to assess the precision of the estimate. All statistical analyses were performed using the specified software, and the results were presented in accordance with standard biostatistical conventions.

## 4. Study Limitations and Future Perspectives

New formulations should be considered in a further study to assess the influence of the disintegrant and filler alone, rather than in combination, to better assess the utility of an intragranular disintegrant. To assess interactions between factors, a quality-by-design approach should be implemented that considers the critical factors identified in this study (type of filler, presence/absence of a disintegrant, and binder concentration). In this regard, in addition to the influence of these factors, their interactions should also be considered.

Several parameters could be varied to improve the Carr Index and interparticle porosity. The Carr Index might be improved by increasing the concentration of the functional excipients in the dispersion, but this also comes with the risk of obtaining tablets that will exhibit extreme values regarding resistance to crushing and will come with the risk of not complying with the disintegration requirements. Decreasing the powder-to-liquid ratio may alter coating formation and could promote a more porous structure, depending on the extent of liquid-induced particle rearrangement and consolidation. Another way to improve results is to change the fillers. Using larger sugar cores may alter particle packing and bulk density characteristics, thereby influencing Carr Index values. Drying conditions may also affect these two parameters, but this may contribute to rougher surfaces. Narrowing the particle distribution might optimise the Carr Index [45,46,47].

The SeDeM expert system can be used to evaluate the compressibility incidence factor, but this factor cannot be confused with the GCI, which comprises all the evaluated parameters that are included in different incidence factors, and as a result, even if the compressibility is low or extremely low, if the other incidence factors tend to outline extremely good results, the GCI will show values higher than the recommended average value, which correlates with its name (good compressibility index might be misleading).

A GCI higher than 5 does not automatically confirm that the excipient is suitable for direct compression, as this index accounts for all five incidence factors. Consequently, the compressibility incidence factor should be checked prior to conducting the direct compression test. Ultimately, GCI should not be viewed as a standalone measure of compressibility, since it combines multiple incidence factors. Although the GCI results exceeded the minimum admitted value of 5, the friability results were in accordance with the pharmacopeial stipulation for E1/E3 (<1%), while for E2/E4 the maximum admitted value was exceeded. The CSFR showed similar results, with higher values for E1/E3 formulations (>200 MPa/%), while values for E2/E4 were below 60 MPa/%. Furthermore, E4 showed the lowest values for crushing strength, tensile strength, and CSFR even though the GCI deprecated the value admitted (5).

As illustrated in Table 4, E2 and particularly E4 achieved GCI values above the SeDeM acceptance threshold despite failing one or more critical mechanical quality criteria. This suggests that the averaging nature of the GCI may partially compensate for deficient individual parameters, masking formulation weaknesses when GCI is interpreted in isolation.

No SEM or image-analysis study was performed. Therefore, the possible presence of twin-core particles cannot be completely excluded, although visual inspection and post-sieving evaluation did not indicate significant core sticking.

Although a DoE approach would have improved robustness, the present study used a stepwise, literature-informed formulation strategy to develop a proof of concept. This limitation is acknowledged, and future work will include DoE-based optimisation where the proposed inputs considered for the experiment will be the presence/absence of the disintegrant, the filler type, and the concentration of Aqua-Polish^®^ STA dispersion at different levels within the outlined range of 15–20 (15, 17.5, and 20%). As a result, two qualitative and one quantitative factor will be considered as inputs, and their influence on the following proposed outputs (particle-size distribution and SeDeM indices (PI, PPI, and GCI), tablet hardness, friability, crushing-strength-to-friability ratio (CSFR), and disintegration time will be evaluated.

Besides the characterisation of the granular excipient, the incorporation of an API might be another point of interest in the future, since one of the research directions of the SeDeM expert system is to outline the amount of a functional excipient needed to improve the compressibility incidence factor, since many APIs need improvements regarding this aspect.

Future work will focus on scaling up the co-processing method, evaluating its robustness under industrial manufacturing conditions, and extending the approach to API-loaded formulations to confirm its applicability to real pharmaceutical products. Additionally, further studies should assess long-term stability and performance across different formulation types.

## 5. Conclusions

This study provides comparative information on binder concentration in the SeDeM diagrams of granular excipients obtained via powder layering onto starter cores. The SeDeM expert system is a method for granule characterisation that addresses flow, compressibility, particle size, and lubricity. These characteristics provide a good basis for optimising excipients to develop a co-processed excipient for further use in the development of conventionally released tablets. Based on the results obtained using SeDeM diagrams, several granular excipients were characterised for use in producing conventionally released tablets. One critical formulation variable considered was the concentration of AquaPolish^®^ STA, which was associated with differences in granule size, with higher concentrations generally producing larger granules.

Although the Carr Index and porosity values were low for all formulations, E3 exhibited the most favourable overall compressibility profile due to its high cohesion index (Icd) and highest GCI. Although E4 exhibited lower mechanical performance than E3, it still achieved GCI values above the acceptance threshold proposed by the SeDeM methodology.

All formulations exhibited excellent flow properties, with E3 and E4 showing the highest flowability incidence factor values. From a lubrication and stability perspective, all four formulations meet the proposed requirements.

Future research should link SeDeM predictions to tablets loaded with the API, compare the new excipients with existing co-processed systems on the market, investigate the scalability of the granulation process, and determine the amount of excipient required to produce uncoated tablets with specific quality properties.

Several aspects of this expert system should be noted: SeDeM is susceptible to multicollinearity and parameter overestimation because it relies on static laboratory measurements that may not reflect modern manufacturing conditions. Moreover, this expert system uses an averaging approach that can mask critical deficiencies. As a result, poor performance in essential properties, such as compressibility, may be obscured by high scores on less critical parameters, leading to misleading suitability assessments.

Within a marketing authorisation, the proposed granules should be designated specifically as a co-processed excipient. If its inclusion in a regulatory dossier is considered, the material’s characterisation should directly reference established general compendial chapters governing raw material testing, such as Powder flow, bulk and tapped density, porosity, etc. The SeDeM evaluation includes all of these and transforms them into a radius for better optimisation. All excipients (new and old) must demonstrate intra- and inter-batch reproducibility, consistent with their intended use. Another aspect that needs to be verified is the physical manufacturing of the co-processed excipient, which must comply with the stipulations of the pharmaceutical pharmacopoeia applied in the pharmaceutical industry.

## Figures and Tables

**Table 1:** The values of the parameters’ characteristics of SeDeM and their radius.

Incidence Factor	Parameter	E1 Value	E1 r	E2 Value	E2 r	E3 Value	E3 r	E4 Value	E4 r
Dimension	Da (g/mL)	0.59	5.90	0.52	5.20	0.61	6.10	0.65	6.50
	Dt (g/mL)	0.66	6.60	0.57	5.70	0.66	6.60	0.72	7.20
Compressibility	ξ	0.17	1.39	0.18	1.53	0.12	0.97	0.17	1.39
	CI (%)	9.92	1.98	9.47	1.89	7.16	1.43	10.77	2.15
	Icd (N)	139.50	6.98	144.70	7.24	209.80	10.00	66.20	3.31
Flowability	HR	1.11	10.00	1.10	10.00	1.08	10.00	1.12	10.00
	α(°)	18.30	6.34	12.97	7.41	7.97	8.41	6.84	8.63
	t″ (g/s)	2.28	8.86	5.32	7.34	Immediate (<1 s)	10.00	Immediate (<1 s)	10.00
Lubricity/Dosage	Iθ	0.09	10.00	0.06	10.00	0.06	10.00	0.04	10.00
	Pf < 160 μm (%)	11.72	7.66	4.15	9.17	0.00	10.00	0.00	10.00
Lubricity/Stability	HR (%)	1.66	8.34	1.46	8.54	2.93	7.07	0.71	9.29
	H (%)	10.75	4.62	8.32	5.84	0.81	9.60	0.57	9.72

**Table 2:** Constituents of the four formulations.

Excipient	Role	Amount (g) (*w*/*w*%)			
		E1	E2	E3	E4
AquaPolish^®^ STA concentration (%)	Binder	15	20	15	20
Core type	Coated core	Sugar	Sugar	Sugar	Sugar
		20	20	20	20
Microcrystalline cellulose	Filler	93	93	-	-
Lactose	Filler	-	-	94	94
Pruv^®^	Lubricant	1	1	1	1
Sodium alginate	Disintegrant	1	1	-	-
Sorbitol	Sweetener	5	5	5	5
Dispersion of AquaPolish ^®^ STA	Binder	1.11	1.18	1.12	1.18

**Table 3:** Factors applied to the parameters to obtain the radius value.

Parameters	Value Range (v)	Radius Range (r)	Factors Applied to Limit Value
D_a_	0–1 g/mL	0–10	10 × v
D_t_	0–1 g/mL	0–10	10 × v
ξ	0–1.2	0–10	(10 × v)/1.2
CI	0–50%	0–10	v/5
I_cd_	0–200 N	0–10	v/20
HR	3–1	0–10	(30 − 10 × v)/2
α	50–0°	0–10	10 − (v/5)
t”	20–0	0–10	10 − (v/2)
%RH	0–10%	0–10	10 − v
%H	20–0%	0–10	10 − (v/2)
P_f_	50–0%	0–10	10 − (v/5)
Iθ	0–0.02	0–10	500 × v

**Table 4:** A summary of GCI and mechanical results of the E1–E4 compacted granules.

Formulation	GCI (>5)	Friability (%)	Friability Compliant?	CSFR (MPa/%)	Notable Observation
E1	Yes	<1	Yes	>200	Acceptable mechanical performance
E2	Yes	>1	No	<60	High GCI despite poor friability and low CSFR
E3	Yes	<1	Yes	>200	Acceptable mechanical performance
E4	Yes	>1	No	<60	High GCI despite friability failure and the lowest crushing strength, tensile strength, and CSFR

## Data Availability

The original contributions presented in this study are included in the article/Supplementary Material. Further inquiries can be directed to the corresponding authors.

## Supplementary Materials

The following supporting information can be downloaded at: https://www.mdpi.com/article/10.3390/ph19091481/s1, Table S1. The PI, PPI and GCI calculated for the proposed granules. Table S2. Descriptive statistics—Tablet thickness. Table S3. Descriptive statistics—Tablet diameter. Table S4. Descriptive statistics—friability. Table S5. Descriptive statistics—resistance to crushing. Table S6. Descriptive statistics—tensile strength. Table S7. Descriptive statistics—CSFR. Table S8. Descriptive statistics—disintegration ability.

## Abbreviations

The following abbreviations are used in this manuscript:

API	Active Pharmaceutical Ingredient
CI	Carr Index
CoPE	Co-Processed Excipient
CSFR	Crushing Strength/Friability Ratio
Da	Bulk Density (g/mL)
DMF	Drug Master File
DoE	Design of Experiments
Dt	Tapped Density (g/mL)
EMA	European Medicines Agency
FDA	Food and Drug Administration
f	Reliability Factor
GCI	Good Compressibility Index
H (%)	Hygroscopicity
HR	Hausner Ratio
%HR	Loss on Drying
Icd	Cohesion Index
ICH	International Council for Harmonisation
IPEC	International Pharmaceutical Excipients Council
Iθ	Homogeneity Index
MCC	Microcrystalline Cellulose
ODT	Orodispersible Tablet
Pf	Percentage of Particles < 160 µm
PI	Parameter Index
PPI	Parameter Profile Index
QbD	Quality by Design
QRM	Quality Risk Management
RH	Relative Humidity
SeDeM	Sediment Delivery Model
t″	Flow Factor
Ts	Tensile Strength
USP	United States Pharmacopeia
USP-NF	United States Pharmacopeia–National Formulary
α	Angle of Repose
ξ	Interparticle Porosity
