# Supplementary material for: Evaluation of Novel Co-Processed Excipients for Direct Compression Using the Sediment Delivery Model (SeDeM) Expert System
Source: Pharmaceuticals (Basel). 2026 Sep 17;19(9):1481. doi: 10.3390/ph19091481 (PMC13609960; doi:10.3390/ph19091481)
Supplement: Supplementary file 1 [file pharmaceuticals-19-01481-s001.zip › pharmaceuticals-4483455-supplementary.pdf]

Article

Formulation and Functional Characterization of a Novel Co-Processed Excipient for Direct Compression: Assessment by the SeDeM Expert System

Adriana Ciurba [1], Paula Antonoaea [1,*], Emőke Margit Rédai [1], Andrada Pintea [1], Cezara Pintea [1], Amalia-Adina Cojocariu [1], Magdalena Bîrsan [2,*], Mădălina-Florentina Mihalcea [1] and Robert-Alexandru Vlad [1]

[1] Pharmaceutical Technology and Cosmetology Department, Faculty of Pharmacy, George Emil Palade University of Medicine, Pharmacy, Science and Technology of Targu Mures, 38th Gheorghe Marinescu Street, 540142 Targu Mures, Romania

[2] Grigore T. Popa University of Medicine and Pharmacy from Iaşi, 16 Universităţii Street, 700115 Iaşi, Romania

* Correspondence: paula.antonoaea@umfst.ro (P.A.); magdalena.birsan@umfiasi.ro (M.B.)

Material and methods:

Table S1. The PI, PPI and GCI calculated for the proposed granules

Index	E1	E2	E3	E4
**PI**	0.75	0.83	0.83	0.75
**PPI**	6.62	6.79	7.64	7.43
**GCI**	6.30	6.46	7.28	7.07

Table S2. Descriptive statistics – Tablet thickness

Parameters	E1	E2	E3	E4
n	3	3	3	3
Mean ± SD	3.735 ± 0.005	3.881 ± 0.001	4.087 ± 0.007	4.088 ± 0.001
Median	3.735	3.881	4.087	4.088
Minimum	3.730	3.880	4.080	4.087
Maximum	3.740	3.882	4.094	4.089
Range	0.010	0.002	0.014	0.002
CV (%)	0.134	0.026	0.171	0.024
95% CI of mean	3.723–3.747	3.879–3.883	4.070–4.104	4.086–4.090

Table S3. Descriptive statistics – Tablet diameter

Parameters	E1	E2	E3	E4
n	3	3	3	3
Mean ± SD	12.08 ± 0.005	12.06 ± 0.015	12.05 ± 0.004	12.03 ± 0.006
Median	12.08	12.06	12.05	12.03

Minimum	12.07	12.05	12.05	12.02
Maximum	12.08	12.08	12.06	12.03
Range	0.010	0.030	0.008	0.012
CV (%)	0.041	0.127	0.033	0.050
95% CI of mean	12.06–12.09	12.03–12.10	12.04–12.06	12.01–12.04

Table S4. Descriptive statistics - friability

**Statistic**	**E1**	**E2**	**E3**	**E4**
n	3	3	3	3
Mean ± SD	0.609 ± 0.001	2.700 ± 0.050	0.549 ± 0.001	1.650 ± 0.100
Median	0.609	2.700	0.549	1.650
Minimum	0.608	2.650	0.548	1.550
Maximum	0.610	2.750	0.550	1.750
Range	0.002	0.100	0.002	0.200
CV (%)	0.16	1.85	0.18	6.06
95% CI of mean	0.6065–0.6115	2.576–2.824	0.5465–0.5515	1.402–1.898

Table S5. Descriptive statistics – resistance to crushing

**Statistic**	**E1**	**E2**	**E3**	**E4**
n	6	6	6	6
Mean ± SD	139.4 ± 1.9	143.8 ± 3.7	209.8 ± 6.8	66.0 ± 1.8
Median	139.3	142.5	209.9	66.1
Minimum	137.0	141.0	200.0	64.0
Maximum	142.0	151.0	219.0	68.0
Range	5.0	10.0	19.0	4.0
CV (%)	1.33	2.54	3.24	2.71
95% CI of mean	137.5–141.4	140.0–147.7	202.7–216.9	64.2–67.9

Table S6. Descriptive statistics – tensile strength

**Statistic**	**E1**	**E2**	**E3**	**E4**
n	3	3	3	3
Mean ± SD	1.969 ± 0.015	1.957 ± 0.031	2.713 ± 0.055	0.856 ± 0.013
Median	1.974	1.953	2.742	0.856
Minimum	1.953	1.928	2.649	0.842
Maximum	1.981	1.990	2.747	0.869
Range	0.028	0.062	0.097	0.027
CV (%)	0.74	1.60	2.03	1.55
95% CI of mean	1.933–2.005	1.879–2.034	2.576–2.849	0.823–0.889

Table S7. Descriptive statistics - CSFR

**Statistic**	**E1**	**E2**	**E3**	**E4**
n	3	3	3	3

Mean ± SD	228.9 ± 1.3	53.3 ± 1.9	382.2 ± 7.8	40.1 ± 2.9
Median	228.7	53.2	385.5	39.4
Minimum	227.8	51.5	373.2	37.7
Maximum	230.3	55.3	387.8	43.3
Range	2.5	3.8	14.6	5.6
CV (%)	0.55	3.60	2.05	7.13
95% CI of mean	225.8–232.1	48.5–58.1	362.7–401.6	33.0–47.2

Table S8. Descriptive statistics – disintegration ability

**Statistic**	**E1**	**E2**	**E3**	**E4**
n	6	6	6	6
Mean ± SD	650.0 ± 17.0	500.0 ± 14.1	386.0 ± 4.2	300.8 ± 14.2
Median	650.0	500.0	386.0	300.5
Minimum	625.0	480.0	380.0	281.0
Maximum	675.0	520.0	392.0	321.0
Range	50.0	40.0	12.0	40.0
CV (%)	2.62	2.83	1.10	4.70
95% CI of mean	632.1–667.9	485.2–514.8	381.5–390.5	286.0–315.7
